# Distributive justice and equity in resource allocation: a temporal analysis of hospitalization costs in indigenous populations in Brazil

**DOI:** 10.1186/s12939-024-02102-w

**Published:** 2024-02-05

**Authors:** Luiz Oscar Machado Martins, Marcio Fernandes dos Reis, Alfredo Chaoubah, Guilhermina Rego

**Affiliations:** 1 Juiz de Fora, MG Brazil; 2 Faculty of Medicine, University of Porto, Porto, Portugal

**Keywords:** Equity, Indigenous populations, Arouca law, Unified health system

## Abstract

**Introduction:**

In Brazil, a country of continental dimensions, the health needs of each region have an impact. In this context and the name of the principle of equity, the SUS organizes actions especially aimed at social groups such as the elderly, children, pregnant women, and indigenous peoples. The concept of justice proposed by John Rawls is one of equity, which is essential to this country.

**Methods:**

This is an ecological, descriptive study, which analyzed hospital spending on cardiovascular diseases in the Unified Health System (SUS) among the indigenous elderly population and other ethnicities/colors in Brazil, between 2010 and 2019.

**Results:**

Hospitalization costs and fatality rates for indigenous populations and other colors/ethnicities, between 2010 and 2019, were evaluated. A reduction in hospitalization costs for the indigenous population and an increase in other populations was observed throughout the historical series, while there was an increase in fatality rates for both groups. A comparison was made between hospitalization costs and the fatality rates of indigenous populations and other colors/ethnicities according to sex, between 2010 and 2019. It was observed that regardless of sex, there are significant differences (p<0.05) between hospitalization costs and fatality rates, with higher costs for patients of other colors/ethnicities and higher fatality rates for the indigenous population.

**Conclusions:**

Hospitalization costs due to cardiovascular diseases in elderly people from indigenous populations were lower compared to other ethnicities in most federative units, which may suggest an unequal allocation of resources or access for this indigenous population to the SUS. Although there is no strong correlation between spending on hospital admissions and fatality rates, it was found that these rates increased between 2010 and 2019, while spending was reduced.

## Introduction

Brazil is home to a large number of indigenous communities and many of them live in areas of great ecological importance, such as the Amazon rainforest. These populations play an important role in environmental preservation in Brazil thanks to their deep knowledge and connection with flora and fauna. Their unique ways of living and occupying a place with traditional wisdom greatly help to expand diversity. It is worth highlighting their importance in the maintenance of biodiversity on the South American continent.

Today, there are 1.7 million indigenous people according to data from the 2022 Census, which represents 0.83% of the total population in Brazil [1]. These communities have a strong understanding of their surrounding ecosystems, having developed complex relationships with plants, animals, and land over thousands of years. As such, they possess valuable knowledge about how to manage and protect these environments sustainably, which has been passed down for generations [2].

Indigenous territories have been a frontier of resistance in the face of capitalist greed, expressed in activities such as mining, logging, monoculture, and livestock farming, among other predatory exploitation practices [2].

It is also important to remember and record the history of the first inhabitants of Brazilian territory, who contributed greatly to the current culture of the nation. Traditional knowledge and culture are of paramount importance in the formation of Brazilian society and contact with these populations may generate a recovery of the knowledge they hold. It is worth highlighting that such populations are especially vulnerable when they exist in smaller numbers in the most urbanized and industrialized regions, such as the Southeast and South [3].

Concerning the challenges encountered by indigenous peoples, with obstacles and inequalities faced in the field of health after over 30 years of living under the 1988 Constitution, the omission and institutional negligence of the Brazilian State in providing care to communities located in areas of difficult access stands out.

According to reports from the Special Secretariat for Indigenous Health (Sesai), we can point out that health care for Indigenous populations is limited and justified by the difficulty of access to their villages, with the aggravating fact that few professionals work in locations far from large centers [4].

Other challenges for these people are land dispossession, property damage, and the exploitation of natural resources. We must highlight the need for public policies that aim to extinguish all attempts to reduce or remove their original rights and put an end to illegal activities on these lands, with interests from the private sector increasing the degree of insecurity within territories and creating conflict, which is especially grave in territories where they live isolated. Funai (National Foundation of Indigenous Peoples) and the recently created Ministry of Indigenous Peoples are public bodies that have the competence to manage an agenda that demands specific knowledge regarding these scenarios and individuals [5].

For the Brazilian reality, it will be a great challenge for the SUS (Unified Health System) to guarantee the universal right to health and, at the same time, protect those in vulnerable conditions. To achieve this, it is necessary to organize service networks targeting specific population groups, taking into account social, biological, economic, and cultural conditions [6].

The creation of the SUS in the 1988 Federal Constitution has as its main premise that the State must provide and guarantee access to health for the entire Brazilian population. On September 19th, 1991, Organic Health Law number 8080 was passed, detailing the functioning of the system.

According to Ordinance 254 of 1/31/2002, the National Health Care Policy for Indigenous Peoples is part of the National Health Policy, making the determinations of the Organic Health Laws compatible with those of the Federal Constitution, which acknowledges specificities and ethnic, cultural, and territorial rights of indigenous peoples [6]. This proposal was regulated by Decree No. 3,156, of August 27, 1999; which provides for the conditions of health care for indigenous peoples, and by Provisional Measure No. 1,911- 8, which deals with the organization of the Presidency and its Ministries, including the transfer of human resources and other assets intended for health care activities from Funai to the National Health Foundation (FUNASA); and by Law No. 9,836/99, of September 23, 1999, which establishes the Indigenous Health Care Subsystem within the scope of SUS [6–9].

The SUS is organized to serve everyone within the national territory. Aith and Scalco highlight that in addition to its general network of services, the system has been working on the construction of strategic policies that aim to contemplate and guide health actions for population groups in vulnerable conditions. These policies can be differentiated into three types, namely (1) health policies aimed at groups in socio-economic-cultural vulnerability; (2) indigenous health care policies; and (3) care policies by life cycle phases [10]. The indigenous population is therefore included in the first type.

A National Conference in November 1986 was a milestone for some definitions in the national health policy and, as a consequence, the construction of a new health system was directly linked to issues of social justice for the most excluded and vulnerable groups, thanks to the inclusion and acknowledgment of rights for Brazilian indigenous peoples, besides the need to meet their specificities in the construction of the new health system that was about to be born. These peoples needed a differentiated inclusion in the system, focusing on the full exercise of their citizenship and all their social, cultural, and territorial rights, with the following justifications:

1. Brazil is one of the countries with the greatest diversity of indigenous peoples in the world, with around 305 ethnicities, different ways of life, languages, social organizations, and cultures;
2. The living and health conditions of these peoples are related to guaranteeing possession and security of their territories, requiring specific articulations with indigenous policies since many groups are threatened by conflicts over land;
3. One of the main aggravating factors in the health situation of indigenous peoples is precisely the contact with national society and State interventions in their territories and ways of life. The logic of public policy operations must recognize these aggravating factors and act with broad consultation and participation of these people to guarantee their protection.

The universality of coverage is something that is progressively being achieved within the objectives established in the Federal Constitution of 1988, and the limiting factor for expanding the effective coverage of SUS services is the allocation of the available amount of financial resources for complete investment and funding.

If, on the one hand, the SUS presents its challenges in guaranteeing equity, universality, and access, on the other, the change in the demographic profile of the Brazilian population, with an increase of older individuals and a reduction of younger ones, promotes changes in the epidemiological profile, with an increase of cardiovascular diseases [11].

It is estimated that this increase in cardiovascular diseases will be greater in older individuals and in countries that are undergoing economic development, as in the case of Brazil [12].

In the last decade in Brazil, these diseases were responsible for the greatest economic impact on SUS hospitalization costs among all the others listed in the International Classification of Diseases, Tenth Revision (ICD-10). However, despite this impact, little is known about its relevance to indigenous populations [13].

The health needs of each region have a huge impact in a continental country such as Brazil and, even considering this context and the principle of equity, the SUS also organizes actions aimed at specific social groups such as older individuals, children, pregnant women, and indigenous peoples. Our main objective is to analyze hospitalization costs and fatality rates from cardiovascular diseases in older individuals from indigenous populations and other ethnicities, in the Brazilian Unified Health System, between 2010 and 2019, and discuss equity relations in resource allocation.

## Materials and methods

This is an ecological, descriptive study, which analyzed hospitalization costs for cardiovascular diseases in the Unified Health System (SUS) among older individuals of the indigenous population and other ethnicities in Brazil, between 2010 and 2019. We have used the administrative database, which is in the public domain and freely accessible, from the SUS Hospital Information System (SIH/SUS), an integral part of the Information Technology Department of the Unified Health System (DATASUS). Data was extracted using the TABWIN software, version 4.5.1.

As inclusion criteria, chapter IX (diseases of the circulatory system) of ICD-10 was selected, which covers diseases related to cardiovascular ones in Brazil, the age group over 60 years of age (only the elderly population), and sex (male and female), organized by Brazilian federative units as follows: Rondônia (RO), Acre (AC), Roraima (RR), Amazonas (AM), Pará (PA), Amapá (AP), and Tocantins (TO), components of the North region; Maranhão (MA), Piauí (PI), Ceará (CE), Rio Grande do Norte (RN), Paraíba (PB), Pernambuco (PE), Alagoas (AL), Sergipe (SE), and Bahia (BA), components of the Northeast Region; Minas Gerais (MG), Espírito Santo (ES), Rio de Janeiro (RJ), and São Paulo (SP), components of the Southeast Region; Paraná (PR), Santa Catarina (SC), and Rio Grande do South (RS), components of the South region; Mato Grosso do Sul (MS), Mato Grosso (MT), Goiás (GO), and the Federal District (DF), components of the Center-West region of the country.

Also as an inclusion criterion, the option “skin color/ethnicity” was selected in the TABWIN software, and data relating to hospitalization costs (relationship between hospitalization costs for cardiovascular diseases in older individuals and the number of hospital admissions recorded) and fatality rate (relationship between the number of deaths from cardiovascular diseases in older individuals and the number of hospital admissions for the same cause) were separated for indigenous populations and those of other ethnicities (white, black, and mixed-race individuals). Diseases recorded by other ICD-10 chapters and that were not specific for individuals over 60 years of age were excluded from the analysis.

Hospitalization costs were obtained in Reais (R$) and converted into US dollars (U$) at a ratio of U$1.00 for every R$3.95 (the average exchange rate of these currencies between 2010 and 2019).

In the bivariate analysis between the “Indigenous” and “Other ethnicity” populations and between the “Male” and “Female” sexes, the non-parametric Mann-Whitney U test was used, with a significance level of 0.05 (*p* < 0.05).

The maps were prepared by the TABWIN software, using the results from the Mann-Whitney U test and the average hospitalization costs and fatality rates for cardiovascular diseases in older individuals for both populations, between 2010 and 2019. The maps were presented in tertiles (33% of the results for each variable) and for each Brazilian federative unit, totaling 27 states.

This research was developed as part of the postgraduate program in Public Health at the Federal University of Juiz de Fora (UFJF) and was exempt from consideration by the Research Ethics Committee since it used data from official health information systems, in the public domain, respecting the principles of National Health Council Resolution No. 466, of December 12, 2012.

## Results

**Fig. 1:**
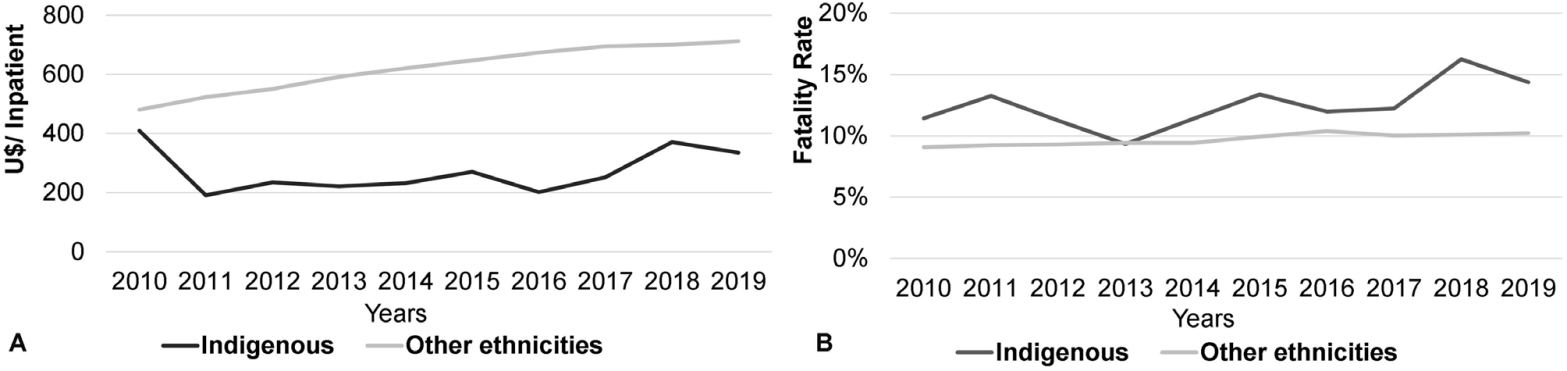
Hospitalization costs and fatality rates due to cardiovascular diseases in older individuals from indigenous populations and other ethnicities in Brazil, between 2010 and 2019. Source: SIH/SUS. Elaborated by the authors. Significantly different results (*p* < 0.05) between “Hospitalization Cost (U$/Inp.)” and “Fatality Rate” of Indigenous populations and those of other ethnicities between 2010 and 2019. Mann-Whitney U Test

Hospitalization costs and fatality rates for indigenous populations and other ethnicities, between 2010 and 2019, are shown in Fig. 1 (A). A reduction in hospitalization costs for the indigenous population was observed throughout the historical series and an increase in other populations, while there was an increase in fatality rates for both groups, in the same period evaluated. It is interesting to note that the values were significantly different (*p* < 0.05) for both hospitalization costs and fatality rates between both groups.

In Table 1, we compared the hospitalization costs and fatality rates of indigenous populations and other ethnicities according to sex, between 2010 and 2019. It was observed that regardless of sex, there are significant differences (*p* < 0.05) between both indexes, with higher costs for patients of other ethnicities and a higher fatality rate for the indigenous population.

**Table 1:** Hospitalization Costs and Fatality Rates due to cardiovascular diseases in older individuals from indigenous populations and people of other ethnicities, according to sex in Brazil, between 2010 and 2019

	Hospitalization Costs (U$/ Inpatient)				Fatality Rate			
	2010	2019	%	*p*	2010	2019	%	*P*
**Male**								
Indigenous	**426**	**396**	-7%	< 0.05	**11%**	**13%**	10%	< 0.05
Other ethnicities	**536**	**788**	47%		**9%**	**10%**	9%	
**Female**								
Indigenous	**329**	**262**	-33%	< 0.05	**11%**	**16%**	44%	< 0.05
Other ethnicities	**425**	**627**	48%		**9%**	**11%**	17%	
**Both sexes**								
Indigenous	**409**	**335**	-18%	< 0.05	**11%**	**14%**	26%	< 0.05
Other ethnicities	**480**	**712**	48%		**9%**	**10%**	13%	

Source: SIH/SUS. Elaborated by the authors. Significantly different results (*p* < 0.05) between “Hospitalization Cost (R$/Inp.)” and “Fatality Rate” of Indigenous and Other Ethnicity populations for males, females, and both sexes. Mann-Whitney U test

**Fig. 2:**
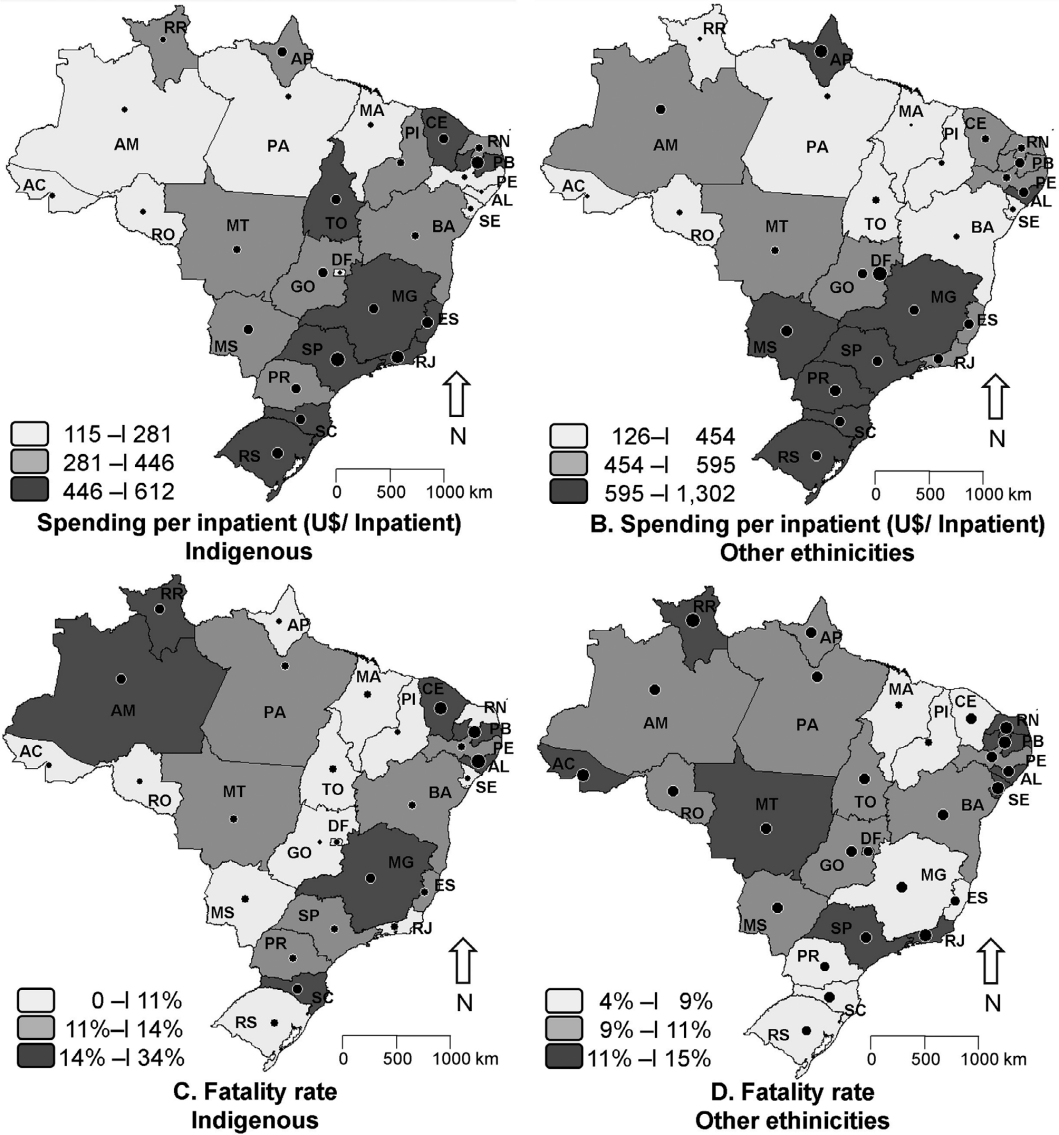
Hospitalization Costs and Fatality Rates for Indigenous People (**A** and **C**) and for Other Ethnicities (**B** and **D**) due to cardiovascular diseases in older individuals, organized by federative units in Brazil. Source: SIH/SUS. Elaborated by the authors

The averages for hospitalization costs and fatality rate for each Brazilian federative unit are shown in Fig. 2. Hospitalization costs (A and B) were higher in the southern and southeastern regions of the country for both groups (RS, SC, SP, and MG) and lower in the northern regions (PA, AC, RO, MA). It can also be observed in A and B that hospitalization costs for cardiovascular diseases in indigenous people presented both a lower and higher limit compared to other ethnicities. In Fig. 2, C, and D, fatality rates did not show a spatial distribution with federative units close to each other, however, a greater range of fatality rates could be observed for the indigenous population, varying between 0 and 34%.

**Fig. 3:**
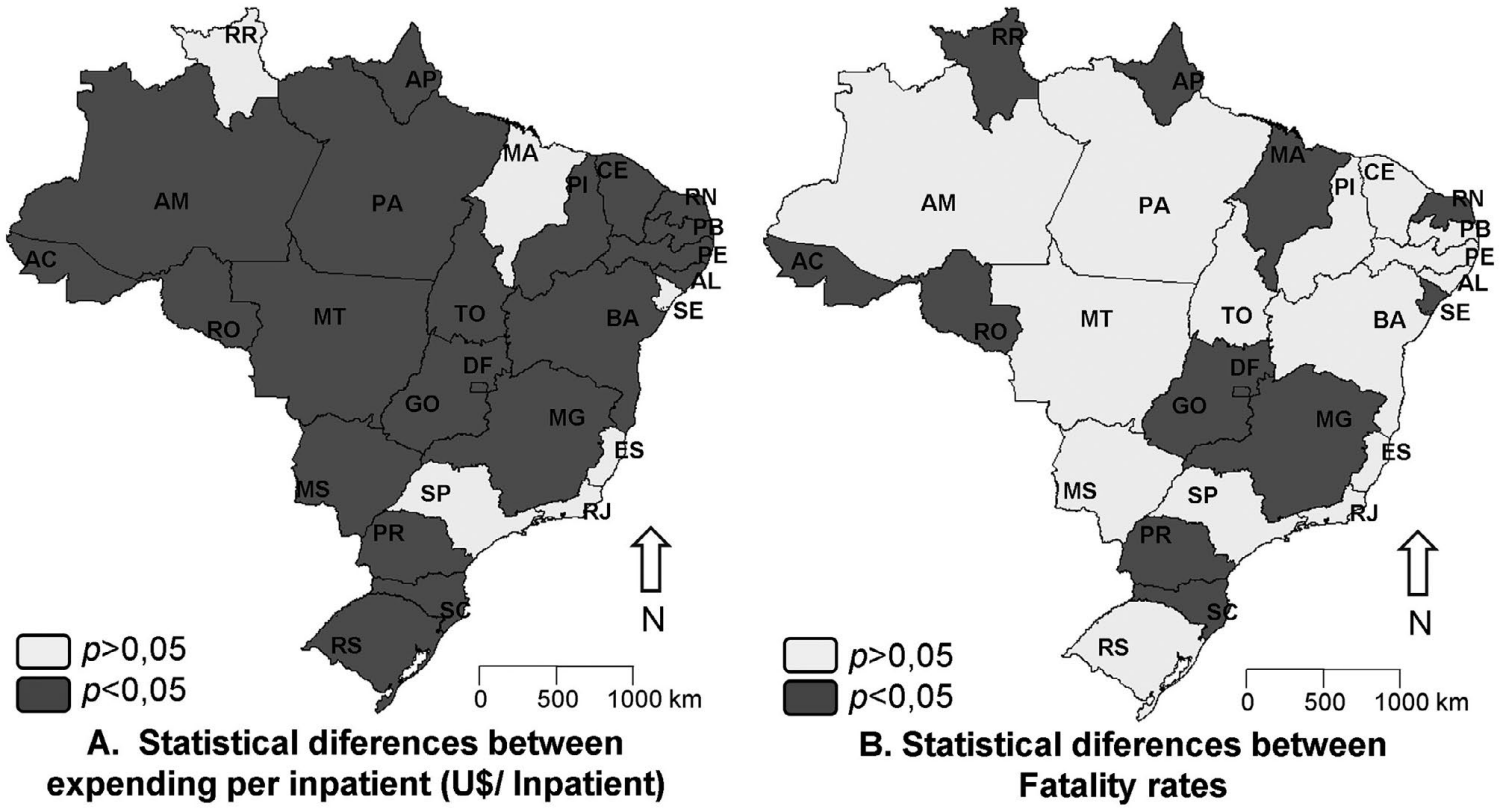
Statistical differences between Hospitalization Costs **(A)** and Fatality Rates **(B)** due to cardiovascular diseases in older individuals from indigenous populations and other ethnicities in Brazil. Source: SIH/SUS. Elaborated by the authors. Significantly different results (*p* < 0.05) between “Hospitalization Costs (R$/Inp.)” and “Fatality Rates” of Indigenous and Other Colors/Ethnicities populations, organized by Brazilian federative units. Mann-Whitney U test

The presence or absence of statistical differences (*p* < 0.05) between hospitalization costs and fatality rates for the indigenous population and other ethnicities are shown in Fig. 3. Regarding hospitalization costs, only the states of RR, MA, SE, ES, RJ, and SP showed no significant differences (*p* > 0.05) in costs between both groups. Regarding fatality rates, 12 federative units showed significant differences (*p* < 0.05) and 15 showed no differences (*p* > 0.05).

Finally, a simple linear regression was performed to try to predict the reduction in fatality rates due to the increase in hospitalization costs. However, linear regression, [F(1,536) = 1.542, *p* > 0.05; R2 = 0.03], did not present results that justified this relationship.

## Discussion

With the Constitution of the Federative Republic of Brazil of 1988, the SUS was established and, according to its article 196, health became a right of all citizens and a duty of the State. In this way, it offers equal and free care and promotion of health for the entire population, covering everything from the simplest to the most complex medical issue, reducing the health inequities that exist between different social groups [14].

The indigenous population in Brazil grew in the North and Central-West regions but decreased in the Southeast and South regions of the country, remaining stable in the Northeast region according to the demographic censuses from 2000 to 2010. With this change in the demographic profile, in which there is an increase in the number of older individuals from indigenous populations in the country, the epidemiological profile also tends to change due to urbanization and the incorporation of new nutritional and lifestyle habits, favoring the emergence of risk factors for cardiovascular diseases [13, 15–17].

In the past, infectious and respiratory diseases, especially acute respiratory failure, were responsible for a large number of hospital admissions and morbidity and fatality among the indigenous population, currently, there may be a change in the epidemiological pattern, with an increase in cardiovascular diseases, due to changes in lifestyle habits, cultural modifications, and increased urbanization [15, 18].

In this line of thought, corroborating the results of this research, it was found that fatality rates due to acute myocardial infarction (an important cardiovascular disease) were higher in the indigenous population, in relation to the rest of the entire population, justified by their being in rural areas, far from access to SUS services [19].

A plausible hypothesis as to why hospitalization costs were lower for the indigenous population may be related to the fact that this group has access to places where the SUS infrastructure is inferior compared to large urban centers [20].

According to Garnelo and Pontes, as a consequence of the sustained and progressive population increase of these groups, several challenges have come to light, especially for the health area, expressed through sensitive transformations in the profiles of illness and death, which can be evidenced due to higher hospitalization costs in the Southeast and South regions for both groups analyzed, despite differences between indigenous populations and those of other ethnicities.

An important national survey promoted by the Ministry of Health and Funasa was a milestone for health in Brazil, as it provides for the first time information on the health and nutrition of indigenous peoples in a nationally representative sample whose collected data will serve as a useful resource for future assessments of the Brazilian Indigenous Health Subsystem and its performance since disparities observed in health indicators highlight that basic health and sanitation services are not yet as widely available in indigenous communities in Brazil as in the rest of the country [16].

There is an unequal relationship between access to health services in populations of low socioeconomic status and ethnic-racial status, as in the case of indigenous populations, which contributes to the reduction of health spending and low resolution of medical issues, in line with the low hospitalization costs for the indigenous population, found in this research [21]. In addition to the difficulty of access that can limit public health actions and spending, the absence of indigenous people included in professional teams; the organizational structure of the SUS itself, sometimes bureaucratic; and the mistaken perception that ethnic-racial inequalities are conceived as a privilege, and not as a search for universalization and equity, can be identified as barriers to the expansion of this system among this population [22].

Indigenous people often have difficulty accessing key primary health services and ensuring access to them requires more than their availability within easy geographical reach [23–24]. Within the scope of the SUS, with the creation of a sub-system with 34 Special Health Districts specific to this population and of the Indigenous Health Care Information System (SIASI), information on demography and morbidity began to be collected more reliably and comprehensively throughout the national territory.

Proof of successful care policies regarding indigenous populations that, as Pontes highlights, “until 1999 were sporadically provided health care services, developed by teams that traveled through indigenous lands providing medical assistance and other specific actions” [21], was the Arouca Law, which became the contextualizing framework for the universality and equality of routines in care directed at these populations.

This law has also guaranteed indigenous peoples access to comprehensive health care, always in accordance with the principles and guidelines of the SUS, contemplating social, cultural, geographic, historical, and political diversity in order to favor overcoming the factors that make this population vulnerable [8].

Discussions about the management of health spending in Brazil raise the question if we are efficient in allocating these resources. To try to clarify this issue, Mendes cites as challenges to improving the health of the indigenous population the historical inequalities existing in our country, the low social participation of this population in political decisions to implement SUS strategies, and ethnic and cultural barriers existing between the way the SUS works and the way of life of these populations [25].

We found that we still allocate fewer resources to this population compared to the other ethnicities in the country, however, Antunes shows that over the years the evolution continued, mainly after the creation of the Special Secretariat of Indigenous Health, whose budget exceeded US$405 million in 2018 [26]. Created in 2010, the secretariat was tasked with coordinating the management of the subsystem, a role it maintains to this day.

The National Indian Foundation (Funai) lists some advances in the implementation of health services for indigenous populations, such as the Creation of the Special Secretariat for Indigenous Health (SESAI), in 2010, and the placement of doctors in villages to provide basic care services in 2013. It points out the challenges of strengthening the basic health care policy, with the installation of health units in all indigenous lands and also high and medium complexity care that respects specificities of this public with the due qualification of care teams that bring a differentiated perspective and respect for their traditional healing practices.

Notorious changes have taken place in the Brazilian public health system since 1988, when the new constitution declared health a universal right. Coelho and Dphil showed that population coverage has grown substantially, and health indicators have improved and, despite these achievements, equity in access continues to be an important barrier to universal coverage, particularly for marginalized groups such as indigenous peoples [27]. To move forward, the innovation cycle needs to be reinvigorated so as to guarantee quality and equity and take advantage of what we have already learned from the implementation processes of the SUS and the Indigenous Health Subsystem.

As a recommendation for excellent and effective care, it is essential that activities related to basic health care for indigenous people are provided, whenever possible, within indigenous lands.

Rawls starts from the conception that the distribution of resources must occur in two stages [2]. In the first, the concern would be with the distribution of basic rights and duties. In the second, based on the principle of difference, unfair inequalities would be compensated, particularly those that affect the most disadvantaged, guaranteeing everyone equal opportunities. Health was conceived by him as a natural primary good and would be a consequence of a fair society.

It is important that the SUS does not become a system dedicated only to the poorest and that it is an important component for the well-being of the entire country thus the long-awaited universality and equality of coverage will depend on a substantial increase in public financing. An increase in the effectiveness of services with a consequent reduction in income inequalities. The indigenous health policy is well-structured thanks to the autonomy of the Special Indigenous Health Districts (Dsei).

The present study has some limitations. The use of secondary public data obtained from the SIH/SUS may present errors in data collection, typing, and storage, which does not prevent DATASUS from continuing to be used as the main source of data available from this system, in addition, other supplementary and private health systems were not included in this analysis [28]. Another issue that deserves to be highlighted refers to the scarcity of national research that analyzes the difficulties faced by indigenous populations in accessing the SUS, the epidemiological profile, and the expenses of the system. Even given the methodological limitations, it is important to highlight that this is the first Brazilian research that analyzes hospitalization costs and fatality rates from cardiovascular diseases in indigenous populations over 60 years of age in Brazil.

As a suggestion, we emphasize the importance of further research on the most vulnerable groups in the country, to draw up a more precise epidemiological diagnosis and provide strategic conditions for the SUS to comply with its principles of equity, integrality, and universality.

## Conclusion

Hospitalization costs due to cardiovascular diseases among elderly people from indigenous populations were lower compared to other ethnicities in most federative units, which may suggest an unequal allocation of resources or access for this population. Although there is no strong correlation between hospitalization costs and fatality rates, it was found that these rates increased between 2010 and 2019, while costs were reduced.

With a better distribution of resources and greater investments in health in this specific population, we can see that we can evolve and improve a lot, maintaining the strong bias pointed out by Rawls of the SUS, where distributive justice with equity for the most vulnerable is already seen in the health policy for indigenous populations [2].

## Data Availability

The data presented in this study are available on request from the corresponding author.
